# Retraction: Thoughts and experiences regarding leg amputation among patients with diabetic foot ulcers: A phenomenological study

**DOI:** 10.1111/iwj.14844

**Published:** 2024-03-14

Retraction: “Thoughts and experiences regarding leg amputation among patients with diabetic foot ulcers: A phenomenological study” by Ka‐Huen Yip, Yuk‐Chiu Yip, Wai‐King Tsui, International Wound Journal 2023, 20: 2159‐2168. The above article, published online on 30 January 2023 in Wiley Online Library (https://onlinelibrary.wiley.com/doi/full/10.1111/iwj.14094) has been retracted by agreement between the authors, the journal Editor in Chief, Professor Keith Harding and John Wiley & Sons, Ltd.

The retraction has been agreed due to major overlap between this article and the following article: Nielsen MK, Bergenholtz H and Madsen UR, Thoughts and experiences on leg amputation among patients with diabetic foot ulcers. International Journal of Qualitative Studies on Health and Well‐being, 2022, Vol 17, 2009202; https://doi.org/10.1080/17482631.2021.2009202. The authors state that the overlap is coincidental and unintentional.

